# Optimization of a Functionally Graded Material Stem in the Femoral Component of a Cemented Hip Arthroplasty: Influence of Dimensionality of FGM

**DOI:** 10.1155/2017/3069351

**Published:** 2017-06-21

**Authors:** Abdellah Ait Moussa, Rohan Yadav

**Affiliations:** Department of Engineering and Physics, University of Central Oklahoma, Edmond, OK 73034, USA

## Abstract

The longevity of hip prostheses is contingent on the stability of the implant within the cavity of the femur bone. The cemented fixation was mostly adopted owing to offering the immediate stability from cement-stem and cement-bone bonding interfaces after implant surgery. Yet cement damage and stress shielding of the bone were proven to adversely affect the lifelong stability of the implant, especially among younger subjects who tend to have an active lifestyle. The geometry and material distribution of the implant can be optimized more efficiently with a three-dimensional realistic design of a functionally graded material (FGM). We report an efficient numerical technique for achieving this objective, for maximum performance stress shielding and the rate of early accumulation of cement damage were concurrently minimized. Results indicated less stress shielding and similar cement damage rates with a 2D-FGM implant compared to 1D-FGM and Titanium alloy implants.

## 1. Introduction

For years, Titanium and Titanium alloys were the preferred materials for orthopedic implants owing to their biocompatibility, excellent corrosion resistance, and their reliable mechanical performance as replacement for hard tissues [1]. Yet, the lack of integration into the bone tissue in addition to implant-host stiffness incompatibility often leads to implant failure [2–5]. Revision surgery to address such failure involves increased risk, complications, and costs. For hip implants, one of the primary causes of failure is the aseptic loosening due to poor bonding and lack of firm fixation of the implant biomaterial to the bone; in this respect, novel cemented fixations and cementless but improved implant designs that incorporate osseointegration with enhanced bioactivity were proven to increase the short- and long-term implant stability and interface bonding strength [6–10]. Stress shielding of the bone owing to stiffness mismatch with the material of the implant is also important and can adversely affect the long-term durability and fixation of the bone-implant construct. Whether cemented or uncemented fixations were used, the stiffer implant shields the load and carrying capacity from the bone leading to reduction in bone density, bone thinning, and bone fracture [11–16].

Stress shielding of the bone can be reduced with careful choice of implant design and material properties and a number of alternative options were investigated. One study revealed that low-modulus Titanium based alloys such as Ti-13Nb-13Zr and Ti-29Nb-13Ta-4.6Zr had effectively reduced stress shielding and provided high levels of biocompatibility [17]; these materials however were susceptible to corrosion and wear and the cost per unit was relatively high [1, 18]. In other studies, reduced micromotion and improved long-term stability were observed in fully cemented fixations [19, 20] and with shorter stem lengths [21]. Alternatively, modifying the implant shape by minimizing the stress concentration on the cement interface or within the cement mantle near the proximal or distal ends of the implant was also considered in a number of studies [22–24] where, despite the diversity of objective functions and numerical schemes employed in these investigations, stress shielding was not part of the design objectives.

It is generally accepted that stiffer stems minimize stress concentration proximally and shield the bone from the mechanical stimulus needed for its internal remodeling. By contrast, softer stems reduce stress shielding and promote higher proximal interface stress, thereby increasing the risk of proximal interface failure. A successful design must therefore allow for a gradual and structured control of the implant stiffness between the proximal and distal ends to reveal the most optimal trade-off in the conflict.

New advances in materials science lead to a novel class of materials known as functionally graded materials (FGMs) where the mechanical properties can be tailored to vary with position within the material by carefully distributing volume fractions of different phases in the gradation directions [25–28]. Power laws and exponential functions are usually used to describe the mechanical properties of the resulting FGMs; these include volume fractions, tensile strength, Poisson's ratio, and modulus of elasticity [29]. A number of studies have demonstrated the suitability of these materials for use in various prostheses including hip, knee, and dental implants [30–36]. Functionally graded materials are investigated in the present study and a material stiffness and volume fraction distributions are proposed to provide the structured composition needed to minimize the integral effects of stress shielding and cement damage.

Most of the studies conducted on FGMs for total hip replacement assumed a simplified two-dimensional geometrical model of the prosthesis, a known FGM composition, or selected three-dimensional implant profiles [37–41]. In the most recent of these studies, a realistic model of a longitudinally FGM (1D-FGM) prosthesis was developed for cemented and uncemented implants [41] where further investigation of the effects of several design configurations upon changing the proximal and distal stem cross-sections along with the gradient index revealed that careful realistic design of the stem together with accurate control of the FGM stiffness distribution can potentially induce more strain energy within the bone and less interface stress with the embedding medium. Nevertheless, a systematic procedure to achieve such level of design synthesis is yet to be developed.

The main purpose of this study is to develop a self-regulated and realistic three-dimensional optimization scheme to accurately design new femoral prostheses using FGMs by carefully controlling the stem geometry and material stiffness distribution in a cemented prosthesis, and the newer designs must effectively reduce stress shielding of the bone without excessively damaging the cemented fixation.

This paper is structured as follows; in Section 2, we review the computational technique used to realistically optimize the geometry and material stiffness of the FGM stem in a cemented hip prosthesis. In Section 3, we apply the methodology thus introduced and identify the optimal stem shape, stiffness distributions, and constituent fractions that equally minimize stress shielding and cement damage. In the final section, we conclude with a summary and discussion of future work.

## 2. The Computational Technique

In this section, we shall describe the general structure of the computational technique used to model and optimize the shape and material stiffness distribution of a cemented bidirectional FGM (2D-FGM) implant.

### 2.1. Solid Modeling of the Implant-Cement-Bone Construct

The mathematical and solid modeling of the cemented prosthesis-femur construct used in the current investigation was introduced by the author and his collaborators in a previous publication [42]; for consistency and flow of information among the different sections of the manuscript, we summarize the major steps of this process.

The implant skeleton was modeled using six cross-sections along its length (i.e., prosthesis and neck axes); the most proximal and most distal cross-sections were kept fixed in size and shape while the other four cross-sections were allowed to vary in size and shape from circular to oval and trapezoidal profiles. The solid model of the resulting implant was then precisely embedded in the intramedullary canal of a femur model where the cancellous bone was omitted for simplification, a cement layer filled the gap between the implant and the cortical bone, and a cemented implant-bone construct was formed. The design process was entirely automated to allow for updates in the parameters of the implant cross-sections.

### 2.2. Modeling of the Functionally Graded Material of the Stem

In order to investigate the mechanical performance of FGMs with varying properties, numerous mechanical and mathematical models were developed, in the homogeneous multilayer (HML) model, for instance [43, 44], the material of the FGM was sliced in the gradation directions, and constant material properties were assigned to each layer. This model was first developed to study the geological media and was later employed in the study of crack problems in FGMs with arbitrarily varying material properties [45, 46]. The spatial distribution of the material properties is discontinuous between the sublayers of this model and a great number of slices may be required to yield sufficiently accurate results. The model is convenient for complex geometries and can also be used in the study of FGMs with continuously varying material properties. In this section, we shall apply the HML model to the functionally graded implant developed in Section 2.1.

**Figure 1:**
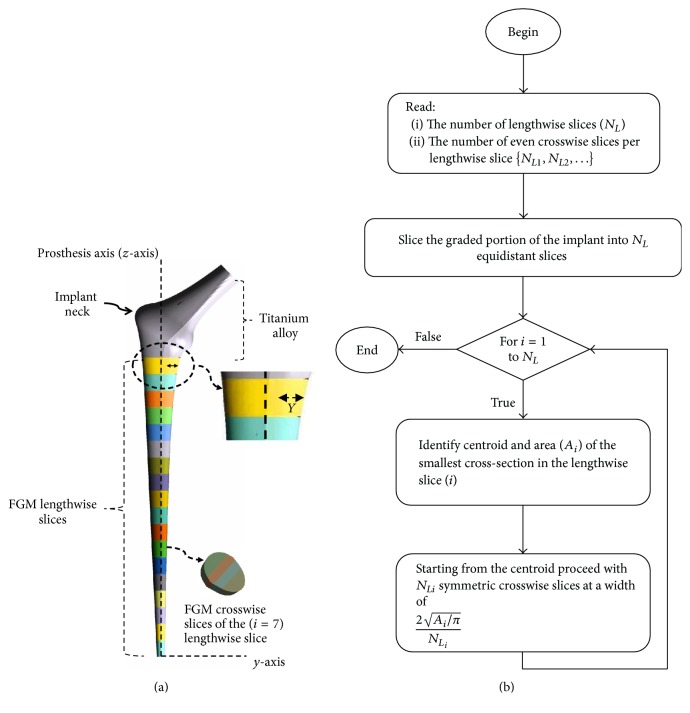
(a) The lengthwise slices were equidistant; the most distal slice was kept intact while the rest were sliced crosswise in a nearly equidistant manner. The crosswise width was computed based on the radius of the smallest cross-sectional area of the lengthwise slice; the crosswise slicing process began at the centroid of the smallest cross-section and symmetrically outward. Even number of crosswise slices were used. (b) Slicing flow chart.

A fixed length in the proximal end of the implant as depicted in Figure 1(a) was assumed homogeneous with material properties identical to that of Titanium alloy (Ti-6Al-4V with modulus of elasticity of 110 GPa and Poisson's ratio of 0.3) [47], the length extended over the proximal medial region of the cement where the most likely sites of failure initiation occur owing to the abrupt change in the implant cross-sections [48], and choosing a stiffer material such as Titanium minimizes the likelihood of failure initiation at these locations. The rest of the implant consisted of a functionally graded material where the modulus of elasticity was decreasing toward the distal end and crosswise toward the lateral side of the implant according to the power law in (1). Insight into devising such a model stemmed from previous research [39, 40] where it was demonstrated that stress shielding and interface shear stress were reduced upon reduction of implant material stiffness from the proximal to the distal end and crosswise toward the lateral side of the implant with a relatively steeper change near the proximal end:

(1) Ez=ELETiELz/L,EY,z=EzEREzY/2r.

**Figure 2:**
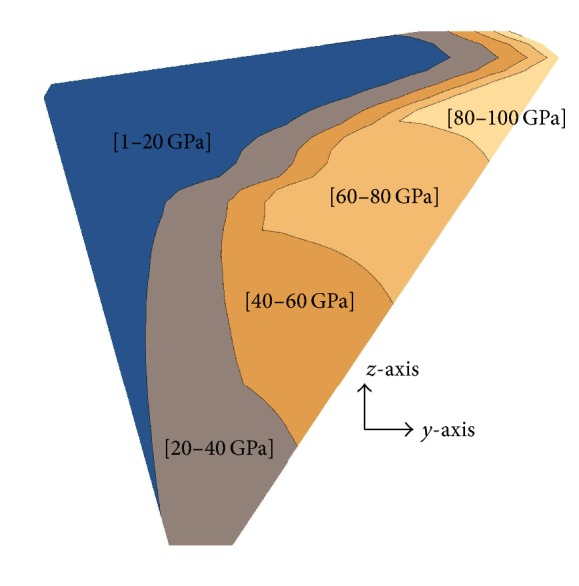
Contour plot of the material stiffness in (1) for one of the implants.

In (1), (*Y*, *z*) are, respectively, the horizontal and vertical distances from the medial side and distal end of the implant as depicted in Figure 1(a), (*L*) is the length of the graded section along the prosthesis axis, (*r*) is the radius of the distal cross-section, (*E*_Ti_) is the modulus of elasticity of Titanium alloy (Ti-6Al-4V), and (*E*_*L*_) and (*E*_*R*_) are control stiffness's such that (*E*_*R*_ ≤ *E*_*L*_ < *E*_Ti_); this condition ensures that *E*(*Y*, *z*) is decreasing toward the distal (smaller {*z*}) and lateral (larger {*Y*}) sides of the implant. A contour plot illustrating the overall material stiffness distribution for one of the implant geometries (*L* = 145 mm, *r* = 6.0 mm, *E*_Ti_ = 110 GPa, *E*_*L*_ = 25 GPa, and *E*_*R*_ = 15 GPa) is displayed in Figure 2.

To assign continuum material properties, the graded section was sliced along the implant's length and crosswise into a fixed number of slices as described in Figure 1(a), and the number of slices increased between the distal and the proximal ends. The modulus of elasticity of each slice was assumed uniform and equal to that at the centroid of the slice, and bonded boundary conditions were assumed between slices.

The slicing process was automated using a self-developed computer program using the scripting language of the ANSYS Workbench-Design Modeler, and the executable java-Python script was called every time design parameters have changed and the prosthesis model updated. The computational slicing process is summarized in a simplified flow chart in Figure 1(b).

### 2.3. Evaluation and Optimization of Stress Shielding and Cement Accumulated Damage

Load is transferred through the implant, cement, and bone via compression, shear, and assembly bending. Excessive shear and/or compressive stress on the cement can cause damage leading to crack formation, eventually separating the implant/cement interface [49, 50]. Moreover, the lack of the mechanical stimulus to bone remodeling owing to minimum strain energy or less compressive stress commonly known as stress shielding can lead over time to excessive bone resorption and bone thinning, eventually loosening the fixation [51]. The relevance and conflicting interests of these factors are equally essential to the long-term durability of the fixation and cannot be prioritized over one another. Nevertheless, a control over the implant rigidity via shape and material stiffness distribution can equitably minimize the likelihood of future fixation failure by simultaneously minimizing the rate of early cement damage, since this can potentially delay implant/cement interface separation, and the difference in the mechanical stimulus with reference to the intact bone to impede bone resorption.

To identify the implant designs and material compositions that achieve these objectives, we used the design based optimization technique developed in a previous research by Ait Moussa et al. [42]. In the current setting, the control parameters were selected as the geometrical design parameters of the four cross-sections in addition to the material stiffness of (*E*_*L*_) and (*E*_*R*_) in (1). The application and results of this optimization are discussed in Section 3.

## 3. Results and Discussion of the Numerical Results 

### 3.1. Optimization Setup

**Figure 3:**
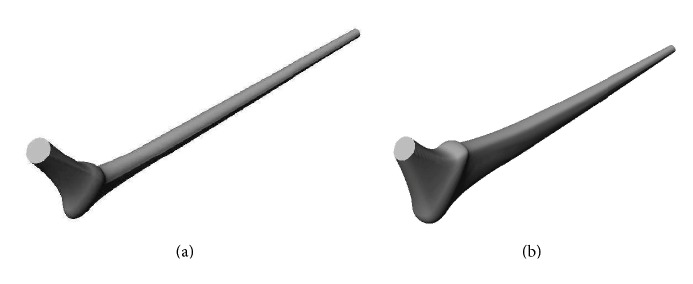
(a) Lowest and (b) uppermost implant geometrical configurations.

The radii of the circular circumferences on both ends of the implant skeleton were identical and set to a value of *r* = 6 mm, the length of the graded section was *L* = 145 mm, the size and profile of the four cross-sections were selected so the produced models were clinically admissible, and the cement thickness is no less than 3.0 mm. The implant configurations corresponding to the lowest and highest geometrical values of the design parameters were, respectively, displayed in Figure 3. Eighteen (*N*_*L*_ = 18) lengthwise slices were produced over the graded section of each implant model and the number of crosswise slices varied from the distal to the proximal end according to the scheme in Table 1; a total of one hundred five slices were accumulated by the end of this procedure. The value of (*E*_Ti_) in (1) was set to equal the modulus of elasticity of Titanium alloy (Ti-6Al-4V: *E*_Ti_ = 110 GPa), and the intervals of variation of 10 GPa ≤ *E*_*R*_ ≤ 20 GPa and 20 GPa ≤ *E*_*L*_ ≤ 60 GPa were selected so the minimum stiffness reported is no less than that of collagen (*E*(*Y*, *z*) ≥ 1 GPa). Parallel computing with ten processor nodes was used in the FEA and in the evaluation of the stress shielding coefficient (*f*_*s*_) and the rate of early cement damage (*f*_*c*_) as described in Ait moussa et al. [42].

### 3.2. Results and Discussion

**Figure 4:**
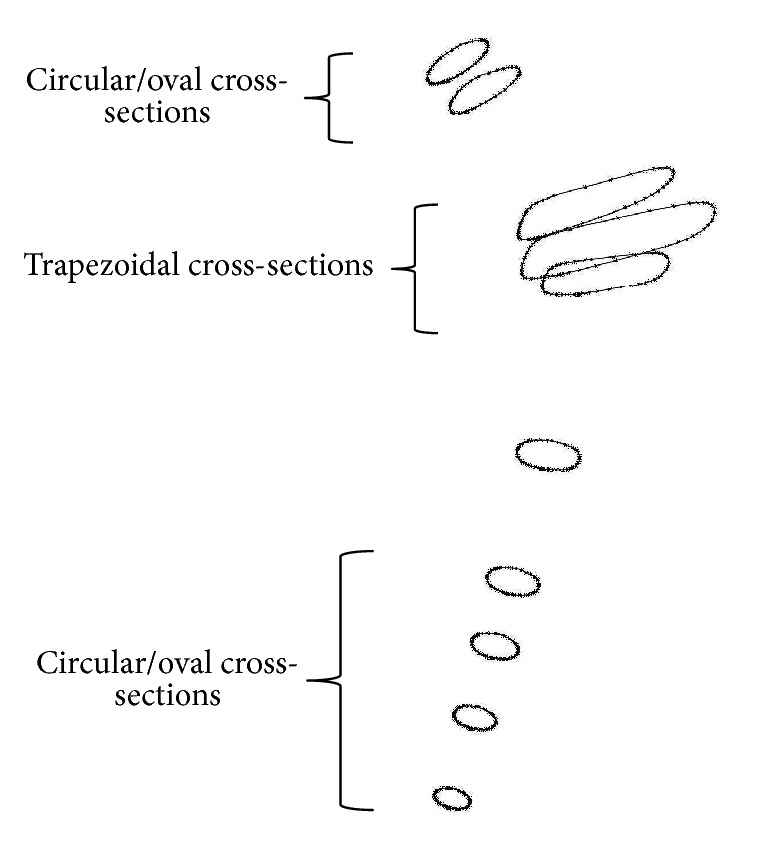
Trapezoidal cross-sections in the central region gradually developing into oval and circular cross-sections near the proximal and distal ends.

Several optimal configurations were identified following the optimization of the implant geometry and material stiffness distribution; nonetheless, the values of the stress shielding and accumulated cement damage coefficients (*f*_*s*_, *f*_*c*_) were not identical between designs. Careful inspection of the results indicated typical trapezoidal cross-sections in the central region of the implant gradually developing into oval and circular cross-sections near the proximal and distal ends as depicted in Figure 4. This result was consistent with previous research [42, 48] where it was demonstrated that a transition to a wider trapezoidal cross-section (from the proximal end) had produced a reduction in stress concentration around the implant which is favorable over the proximal cement since it would minimize the likelihood of failure initiation over the implant-cement interface. Additionally, the modulus of elasticity at the distal and proximal lateral regions was small overall owing to the smaller *E*_*L*_ and *E*_*R*_ values which despite their disparity among optimal designs were all within 20 GPa ≤ *E*_*L*_ ≤ 27 GPa and 10 GPa ≤ *E*_*R*_ ≤ 20 GPa. Moreover, implants with slender stems and relatively smaller *E*_*L*_ and *E*_*R*_ values had on average smaller stress shielding coefficients and moderate to high levels of cement damage. On the contrary, implant with larger medial to distal cross-section and relatively larger *E*_*L*_ and *E*_*R*_ values had more stress shielding and less cement damage.

A physical interpretation of the process that led to these results can be presented as follows. The prosthesis-bone constructs with the softer implant material (smaller *E*_*L*_ and *E*_*R*_) and slender stem cross-sections were relatively less resistant to bending upon application of the body and abductor muscle forces which led to an increase in the strain energy density of the bone, hence less stress shielding. The expanded flexibility however was in general not in favor of delaying early development of cement damage since it had caused more stress concentration on the proximal cement causing an increase in the accumulated cement damage. A trade-off was achieved during the optimization process where moderate values of stiffness of (*E*_*R*_, *E*_*L*_) and relatively wider trapezoidal cross-sections over the proximal medial region in addition to the circular and oval cross-sections near the distal and proximal ends were selected for the majority of the optimal implant designs to balance between the levels of stress shielding and cement damage.

**Figure 5:**
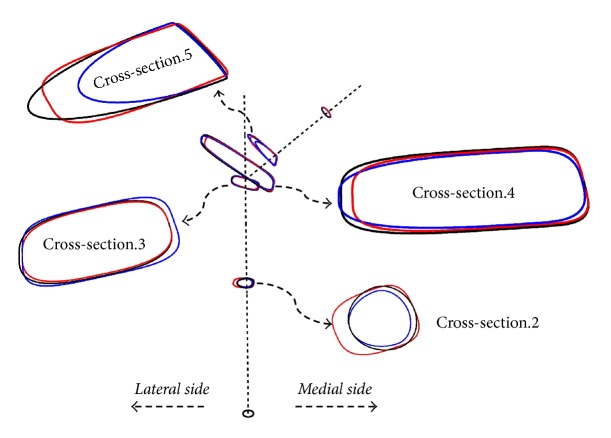
Cross-sectional skeleton of the three optimal configurations. Configuration (a): blue, configuration (b): black, and configuration (c): red.

To elaborate on these results, we selected three implants from the pool of optimal configurations; the respective cross-sectional skeletons are displayed in Figure 5. The cross-sectional profiles (i.e., oval/trapezoidal), stiffness values (*E*_*L*_, *E*_*R*_), and respective stress shielding and accumulated cement damage coefficients are reported in Table 2. The assembly with implant configuration (a) was more susceptible to bending owing to the smaller values of (*E*_*L*_, *E*_*R*_) and the slimmer implant distal cross-sections (cross-section.2) which explains the relatively smaller stress shielding coefficient (*f*_*s*_); more stress concentration however was registered over the proximal implant-cement interface that led to the relatively larger value of the cement damage coefficient (*f*_*c*_). Configuration (c) was slightly more resistant to bending due to the relatively larger values of (*E*_*L*_, *E*_*R*_) and the wider distal stem cross-sections (cross-section.2) which explains its relatively larger stress shielding and slightly smaller cement damage coefficients when compared to configuration (a). Configuration (b) had the least cement damage and the next most optimal stress shielding coefficient; it is by far the most optimal configuration of all three designs. The relatively moderate stiffness values (*E*_*L*_, *E*_*R*_) and narrow distal cross-sections (cross-section.2) were responsible for the reduced bending resistance of the implant-bone construct, hence the relatively smaller stress shielding coefficient; the wider trapezoidal cross-sections over the proximal medial region had uniformly dispersed the stress from the oval cross-sections of the proximal end of the implant and more efficiently than the trapezoidal to trapezoidal cross-sections between the proximal and central medial region of the implant configuration (c) which explains its relatively smaller value of the accumulated cement damage.

**Figure 6:**
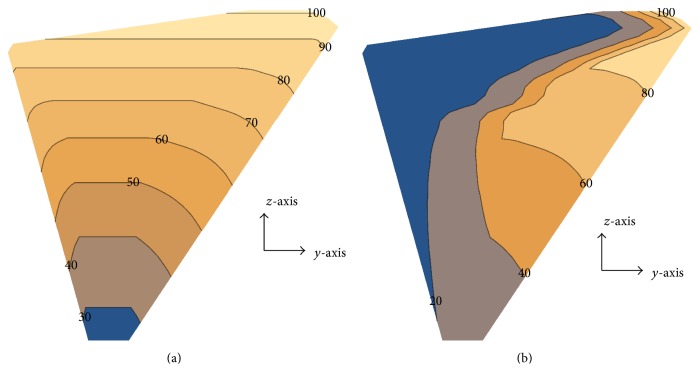
Contour plot of the material stiffness distribution for the (a) 1D-FGM and (b) 2D-FGM.

Additionally, we compared the strain energy density within the femur bone as a function of position along the prosthesis axis when a uniform Titanium alloy (Ti-6Al-4V), a 1D-FGM, and a 2D-FGM implants with identical geometries were used. The most optimal configuration (configuration (b) in Table 2) was selected for this operation and the lengthwise slices in the 1D-FGM were assumed to have uniform stiffness identical to that of the foremost medial crosswise slice in the 2D-FGM implant as depicted in the contour plot of Figure 6.

**Figure 7:**
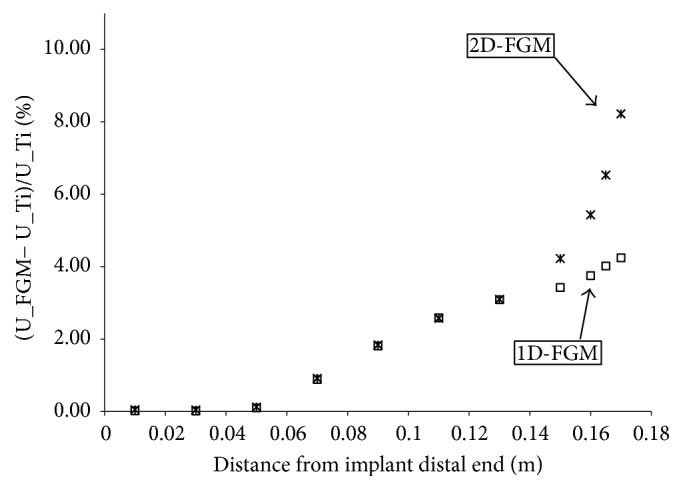
Rate of change of the strain energy density in the femur with reference to the Titanium alloy implant configuration. (U_FGM) is strain energy density in the femur with FGM implant, (U_Ti) is strain energy density in the femur with Titanium alloy implant.

In Figure 7, we represent, over several intervals along the femur axis, the ratio of the change in the strain energy density of the bone with FGM implant to that with a Titanium alloy implant. There is an overall increase in the strain energy density over the medial and proximal regions as indicated by the positive slopes; the 2D-FMG performed better over the proximal region of the femur owing to the premium flexibility of the material of the implant which allowed for additional bending.

**Figure 8:**
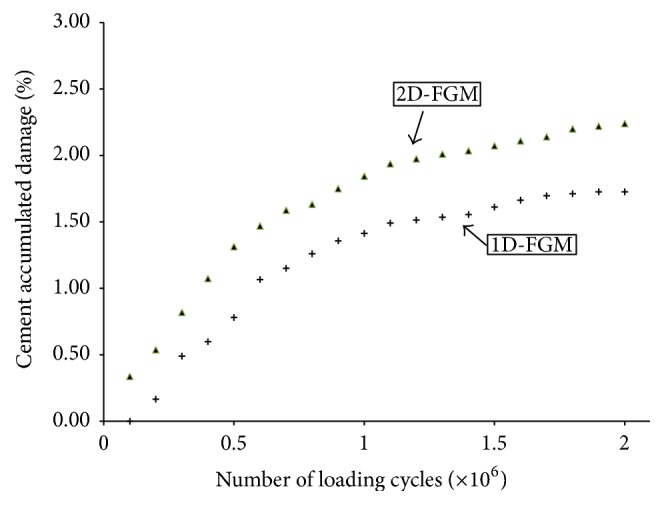
Cement accumulated damage (*f*_*c*_) versus the number of loading cycles.

**Figure 9:**
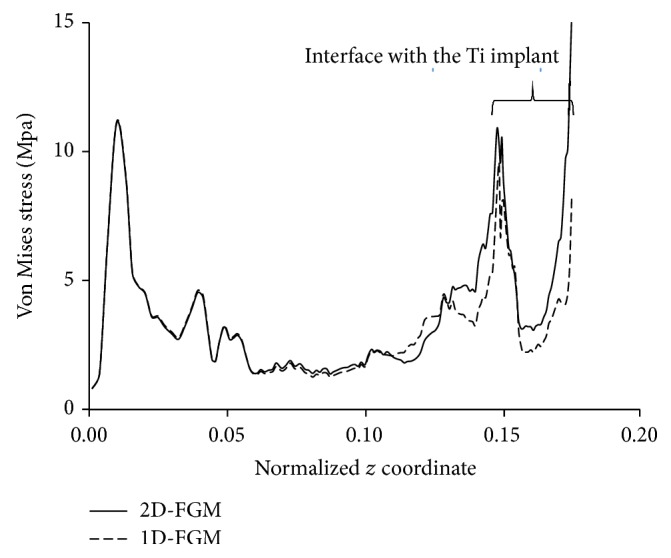
Stress distribution over the cement interface with the implant.

**Figure 10:**
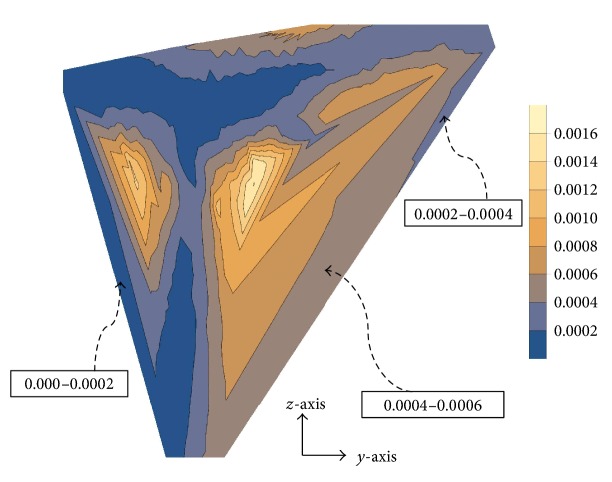
Contour plot of the equivalent strain in the graded portion of the 1D-FGM stem.

**Figure 11:**
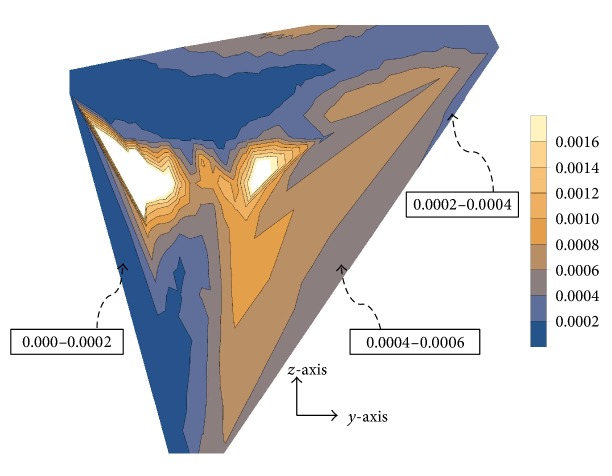
Contour plot of the equivalent strain in the graded portion of the 2D-FGM stem.

The cement accumulated damage (*f*_*c*_) was also calculated at different loading cycles for the cemented 1D- and 2D-FGM implant-bone constructs; the results are displayed in Figure 8. Early cement damage accumulation begins with the 2D-FGM implant and increases at a fairly comparable rate to the implant-bone construct with the 1D-FGM implant. The earlier appearance of cement damage can be explained using the diagram of Figure 9, where the distribution of the Von-Mises stress over the implant-cement interface was plotted as a function of the position from the distal end of the implant. The stress levels with the 2D-FGM implant were noticeably higher over the interface with the Titanium portion of the implant which explains the earlier development of cement damage. The contour plots of Figures 10 and 11 on the other hand indicate relatively similar equivalent strains over the interfaces with the cement, potentially causing equivalent amounts of additional damage in subsequent loading cycles, hence the comparable rates of cement damage accumulation between the 1D- and 2D-FGM implant-bone constructs.

Overall, the benefits of using a 2D-FGM implants in cemented total hip replacement is twofold. On one hand, the additional flexibility of the implant-bone construct improves the strain energy density of the bone upon bending which is essential for its remodeling adaptation. On the other hand, the 2D-FGM implants tend to maintain relatively smaller strains with the cement interface which is essential for reducing the damage accumulation in the cement, hence preserving the life of the implant fixation.

Finally, (1) can be conveniently transformed to indicate the volume fractions of the different constituents of the FGM. To get this result, we applied the logarithm to both sides of the stiffness equation (1) which after manipulation of the different terms reduced to the expression in (2). In a logarithmic stiffness scale, the expressions in brackets can be interpreted according to the linear rule of mixture as the volume fractions of the FGM constituents. In this setup, the graded portion of the implant is made a large fraction of the material with modulus of elasticity (*E*_*L*_) in the distal medial region, a large fraction of the material with modulus of elasticity (*E*_Ti_) in the proximal medial region, and a large fraction of the material with modulus of elasticity (*E*_*R*_) in the distal lateral region:

(2) log⁡EY,z=1−zL1−Y2rlog⁡EL+Y2rlog⁡ER+zL1−Y2rlog⁡ETi.

## 4. Conclusion

We introduced a novel methodology for realistically producing designs of cemented functionally graded hip implants; the self-regulated optimization technique assesses the amount of stress shielding on the bone concurrently with the induced damage rate on the cement and adjusts the geometry and FGM stiffness distribution accordingly to maximize the durability of the fixation. Through the application of the technique, it was demonstrated that changing the flexural strength of the implant through geometrical optimization of cross-sections and the use of functionally graded materials must balance between two conflicting effects; on one hand, cement damage is more pronounced in early loading cycles and preventing early damage accumulation will delay crack development in the cement promoting the stability of the fixation. On the other hand, good prosthesis-bone integration reduces the rate of bone resorption and the likelihood of debonding owing to stress shielding.

Application of the technique also indicated the improved suitability of 2D-FGM compared to 1D-FGM implants in improving the bending flexibility of implant-bone construct and increasing the levels of mechanical stimulus to healthy bone remodeling; additionally the 2D-FGM implant maintained similar levels of equivalent strain with the cement interface which resulted in comparable rates of cement damage accumulation.

Finally, the introduced methodology could be used to investigate additional effects such as the change in implant length and surface structure and be modified to include crack propagation models to accurately assess the life of the fixation; it can also be extended to other orthopedic joint implants such as knee and shoulder implants and to dental implants as well.

## Figures and Tables

**Table 1:** Slicing scheme.

Index of lengthwise slices (*i*)	1	2–6	7–12	13–18

Number of crosswise slices	1	4	6	8

**Table 2:** Cross-sectional profile, FGM stiffness's parameters, stress shield, and cement accumulated damage at the design life of three optimal configurations.

Optimal configurations	Exponents				Stiffness		Accumulated cement damage: *f*_*c*_ (%)	Stress shielding coefficient: *f*_*s*_ (%)
	Cross-section.2	Cross-section.3	Cross-section.4	Cross-section.5	*E*_*L*_ (GPa)	*E*_*R*_ (GPa)		
(a)	Oval	Trapezoidal	Trapezoidal	Oval	22.4	11.2	7.0	27.2
(b)	Oval	Trapezoidal	Trapezoidal	Oval	26.2	18.2	2.2	28.1
(c)	Trapezoidal	Trapezoidal	Trapezoidal	Trapezoidal	26.2	20.0	4.1	30.3
